# Four points regarding reproducibility and external statistical validity

**DOI:** 10.1111/jebm.12498

**Published:** 2022-10-17

**Authors:** Gregory R. Guerin

**Affiliations:** ^1^ School of Biological Sciences University of Adelaide, North Terrace Adelaide South Australia Australia

**Keywords:** BNT162b2, COVID‐19, external statistical validity, reproducibility

Walter et al.^1^ present Phase I–III trial data that show two doses of the BNT162b2 (Pfizer–BioN‐Tech) COVID‐19 vaccine were safe and effective in children aged 5−11 years. Given that millions of children in this age group are receiving the pediatric Pfizer COVID‐19 vaccine, that there are potential risks, and that the balance of benefits over potential risks is more limited in children compared to adults due to low rates of serious disease,^2^ gold standards ought to be applied to supporting data in terms of placebo‐controlled disease endpoint efficacy trials, safety databases large enough to detect adverse events, and appropriate data sharing to enable reproduction and scrutiny of results. Four points are worthy of attention regarding the reproducibility and external statistical validity of the analysis reported in Walter et al.^1^ “External validity” refers to the extent to which conclusions drawn from the data (and statistical tests thereof) are likely to correspond to, or be generalizable to, the real world.^3^ “Reproducibility” refers to the ability of independent researchers to draw the same conclusions from the data.^4^

## 1 SAMPLE SIZE OF SAFETY DATABASE

A total of 2268 participants received injections: 1517 BNT162b2 and 751 placebo.^1^ No myocarditis or pericarditis were reported, a finding Walter et al. state is consistent with low frequency of these events in real‐world data. The supporting citation^5^ reports a rate of 2−3 cases of myocarditis/pericarditis per million doses. Several other studies have found higher rates of these events in young males, indeed their occurrence in this group has been stated to be approximately two orders of magnitude higher, with estimates of 1.1 per 10,000,^2^, ^6^ 1 per 6637^7^ or as high as 1 per 3000.^8^ Indeed, a recent study of 301 13‐ to 18‐year‐old adolescents reported cardiovascular manifestations in 29% of subjects following BNT162b2 vaccination, including seven cases of confirmed or suspected myopericarditis.^9^ Walter et al. report a low 0.14 probability of detecting an event (of any nature) that has a real rate of 1 in 10,000 with 1500 subjects (see Protocol document). UK Government guidance suggests a “safety database of at least 3,000 study participants vaccinated” (see https://www.gov.uk/government/publications/access‐consortium‐alignment‐with‐icmra‐consensus‐on‐immunobridging‐for‐authorising‐new‐covid‐19‐vaccines, accessed 14 December 2021). While there are practical and ethical limits to the feasible size of safety databases in novel trials,^10^ these limitations should be stated clearly in the context of previous assembly of larger safety databases, for example Pellegrini et al.^11^ (>20,000 subjects) and Polack et al.^12^ (>40,000 subjects).

## 2 DISEASE ENDPOINT EFFICACY DATA DO NOT DEMONSTRATE NET BENEFIT

Walter et al. present data on the relative prevalence of symptomatic disease (i.e., COVID‐19) in 5‐ to 11‐year‐olds participating in the trial. The Protocol states that a minimum of 22 cases needed to be detected in the surveillance period to achieve a power of 70%, below which hypothesis testing was not to be conducted. The few cases of COVID‐19 detected during the 5‐ to 11‐year‐old trial did not meet this power criterion, as just 16 cases of symptomatic COVID‐19 were detected during the surveillance period in the placebo group (∼2% of subjects), compared to three in the vaccine group. These case rates represent an absolute risk reduction of just 2%, which does not demonstrate a balance of benefits over risks in terms of relative frequency and severity of disease symptoms versus adverse events. Children usually have mild COVID‐19 symptoms^13^ while close to a third of young vaccine recipients may experience cardiovascular events, with a report of 2% showing elevated cardiac biomarkers or positive lab assessments.^9^ Additionally, the study addressed neither prevention of asymptomatic infection nor subsequent transmission in children, as only symptomatic infection was recorded in the trial.

## 3 EFFICACY SURVEILLANCE PERIOD

Walter et al. calculate efficacy based on surveillance periods of 322 person‐years (treatment) and 159 person‐years (control), respectively, or the equivalent of approximately three months per participant, and report VE of 90.7%. Antibody titers are expected to peak during this period (though correlates of protection are unknown), following which there is a well‐documented decline in BNT162b2 efficacy.^14^, ^15^, ^16^ The reported VE, then, can be applied only to this immediate post‐vaccination period, indeed only to the specific surveillance period. Natural immunity conferred via past infection is prolonged^17^ and therefore risk reduction and potential benefits of vaccination decline over the course of the pandemic, while potential harms remain.

## 4 REPRODUCIBILITY

It is in the public interest that the analytical data used to support peer‐reviewed publication of a clinical trial are shared early (with appropriate protections^18^, ^19^ ^)^. Sharing data allows findings to be independently reproduced, which is an important component of the scientific process^4^, ^20^ and supports integration of studies that did not reach adequate power individually.^19^ The analytical data reported in Walter et al. are available subject to vetted requests 2 years after the study ends (estimated completion date May 2024; https://clinicaltrials.gov/ct2/show/NCT04816643, accessed September 13, 2022). This embargo period represents an “unacceptable delay”^21^ and prevents independent researchers from scrutinizing or reproducing the conclusions made from the data in the meantime.^22^

## 5 CONCLUSION

The consequential findings of Walter et al. cannot be immediately independently reproduced because supporting analytical data will not be released for 24 months following study completion. While withholding data in this manner is not unusual, it prevents scrutiny that should be encouraged where findings have consequences for millions of young children over the intervening period. The external validity and generalization of the findings may be limited by the relatively small size of the safety database, the small number of recorded symptomatic cases in the RCT and the immediately post‐vaccination timeframe of the efficacy data, meaning that reported VE cannot be generalized through time given observed waning of real‐world efficacy. The emergence of the Omicron variant following publication of this study casts further doubts on real‐world vaccine efficacy given the variant has a number of mutations linked to antibody escape and increased transmissibility.^23^
